# Metastasis of Hepatocellular Carcinoma in the Pouch of Douglas Successfully Treated by Radiation Therapy: A Case Report

**DOI:** 10.3390/life13010225

**Published:** 2023-01-13

**Authors:** Hirayuki Enomoto, Masayuki Fujiwara, Hiroshi Kono, Yasukazu Kako, Motonori Takahagi, Junichi Taniguchi, Eri Ishikawa, Naoto Ikeda, Tomoyuki Takashima, Yukihisa Yuri, Nobuhiro Aizawa, Mamiko Okamoto, Kohei Yoshihara, Ryota Yoshioka, Shoki Kawata, Shogo Ota, Ryota Nakano, Hideyuki Shiomi, Takashi Nishimura, Seiichi Hirota, Koichiro Yamakado, Hiroko Iijima

**Affiliations:** 1 Division of Hepatobiliary and Pancreatic Disease, Department of Internal Medicine, Hyogo Medical University, Mukogawa-cho 1-1, Nishinomiya 663-8501, Hyogo, Japan; 2 Department of Radiology, Hyogo Medical University, Nishinomiya 663-8501, Hyogo, Japan; 3 Department of Surgical Pathology, Hyogo Medical University, Nishinomiya 663-8501, Hyogo, Japan

**Keywords:** hepatocellular carcinoma, tumor rupture, peritoneal metastasis, radiation therapy

## Abstract

Metastasis of hepatocellular carcinoma (HCC) in the pouch of Douglas is relatively rare. A 65-year-old man with liver cirrhosis was admitted for detailed examination of a pelvic tumor. He had a previous history of ruptured HCC, and received emergent hemostasis with transcatheter arterial embolization followed by curative ablation. His blood tests showed an increase in des-gamma-carboxy prothrombin (DCP). Contrast-enhanced computed tomography (CE-CT) revealed a heterogeneously enhanced large pelvic tumor, but no additional tumorous lesions were detected in other organs, including the lungs, liver and abdominal lymph nodes. The colonoscopy showed compression by an extra-luminal/submucosal tumor, and computed tomography-guided percutaneous needle biopsy revealed that the pelvic tumor was metastasis of HCC. Because of the poor liver function, the solitary pelvic tumor was treated with three-dimensional conformal radiation therapy (3D-CRT). The tumor size and the DCP value were markedly decreased after radiation therapy. Nine months later, occasional mild bloody stool due to radiation proctitis was observed; however, no serious side effects occurred. Our case suggests that radiation therapy may be a therapeutic option for a solitary metastatic lesion of HCC in the pouch of Douglas.

## 1. Introduction

Peritoneal dissemination is a relatively rare complication of hepatocellular carcinoma (HCC), which was reported to be observed in 2–15% of HCC cases [1]. Tumor rupture, diaphragmatic invasion, and lymph node metastasis were suggested to be risk factors for peritoneal dissemination [2,3]. No decisive treatment protocol for the disseminated tumor has been established, though systemic chemotherapies could be generally selected as a first-line therapy [4,5]. We herein report a case involving a patient who suffered from a large metastatic lesion of HCC in the pouch of Douglas, which was successfully treated with radiation therapy (RT).

## 2. Case Report: Successful Radiation Therapy for the Treatment of Metastasis of Hepatocellular Carcinoma in the Pouch of Douglas (Table 1)

**Figure 1:**
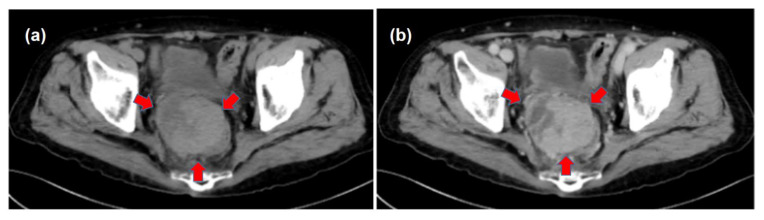
Computed tomography (CT) findings. (**a**) A large pelvic mass of heterogeneous density was detected by plain CT (arrows). (**b**) The pelvic tumor was mildly enhanced on contrast-enhanced CT.

**Figure 2:**
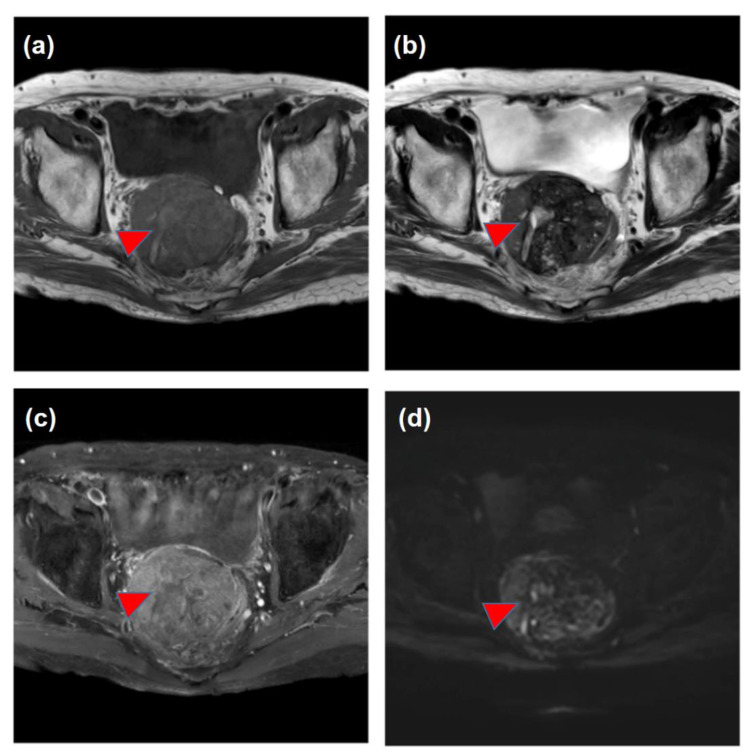
Findings of magnetic resonance imaging (MRI). The pelvic tumor was shown as a hypo-intensity mass both on T1-weighted imaging (**a**) and on T2-weighted imaging (**b**). The tumor showed mild enhancement (**c**), and was detected as a high-intensity lesion on diffusion-weighted imaging (DWI) (**d**). The tumor included heterogeneous lesions, presumably showing necrotic tissue with internal bleeding (arrowheads).

A 65-year-old man with liver cirrhosis was admitted to our hospital for the detailed examination of a pelvic tumor. There were no complaints of any particular symptoms, but periodic blood tests showed an increase in the des-gamma-carboxy prothrombin (DCP); however, no liver tumor was found on abdominal ultrasonography. Contrast-enhanced computed tomography (CE-CT) showed a large pelvic tumor (Figure 1). No other tumorous lesions were detected in the other organs, including the liver, lungs, and abdominal lymph nodes. Contrast-enhanced magnetic resonance imaging (CE-MRI) suggested the presence of a malignant tumor (Figure 2).

**Table 1:** The timeline of the case.

Year	Month	Event
X-5		The diagnosis of NASH-related liver cirrhosis
	January	Rupture of HCC and emergent hemostasis with TAE
X-3	May	Curative treatment of the HCC (re-TAE and additional RFA)
		(Start of HCC follow-up in our hospital after curative therapy)
	March	Increase in the DCP and detection of the pelvic tumor Medical interview revealing mild constipation
X	May	The histological diagnosis of pelvic metastasis of HCC
	August–October	3D-CRT (prescribed dose for the gross tumor: 60 Gy in 30 fractions)
		(Decrease in the tumor size and DCP values, constipation resolved)
X + 1	July	Occasional bloody stool and the diagnosis of radiation proctitis (grade 1) by colonoscopy

HCC: hepatocellular carcinoma; TAE: transcatheter arterial embolization; RFA: radiofrequency ablation; DCP: des-gamma-carboxy prothrombin; 3D-CRT: three-dimensional conformal radiation therapy.

**Figure 3:**
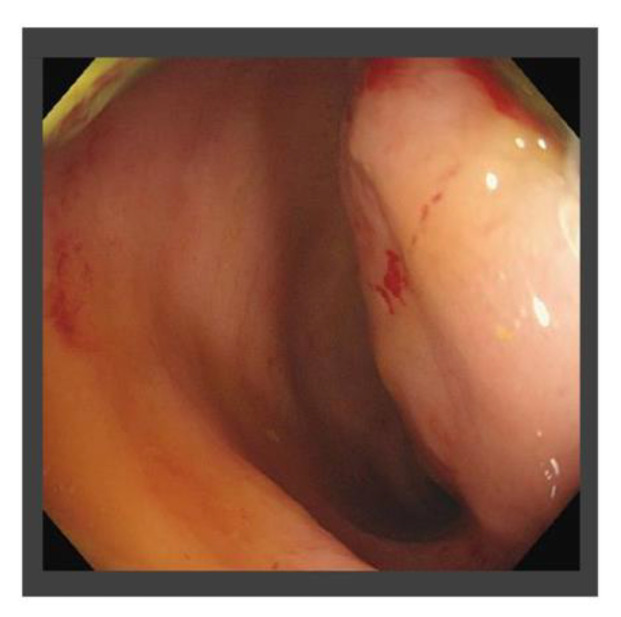
Findings of colonoscopy before radiation therapy. Rectal compression by an extra-luminal tumor was observed, but the tumor did not invade the rectal lumen.

**Figure 4:**
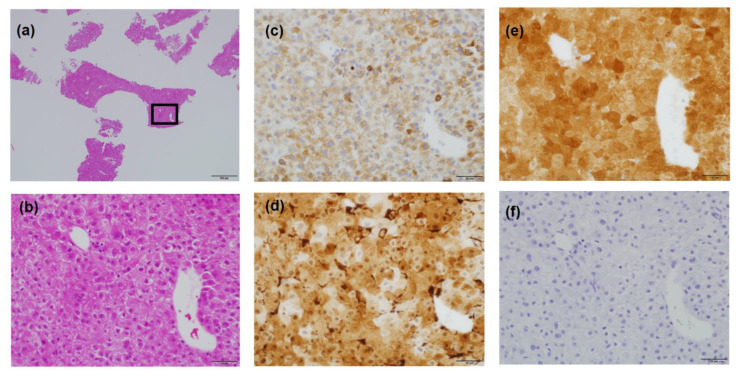
Histological findings of the pelvic tumor. (**a**,**b**) Hematoxylin and Eosin staining. (**a**) A low magnification view of a tumor tissue specimen (×40). (**b**) A high magnification (×400) view of the boxed area in (**a**). The proliferation of malignant cells with acidophilic cytoplasm were observed. (**c**–**f**) Immunostaining of tumor tissue was positive for HepPar-1 (**c**), HSP70 (**d**) and Arginase-1 (**e**). However, in agreement with the blood test results (Table 1), immunostaining of α-fetoprotein was negative (**f**). Bars, 500 μm (**a**) and 50 μm (**b**–**f**).

**Figure 5:**
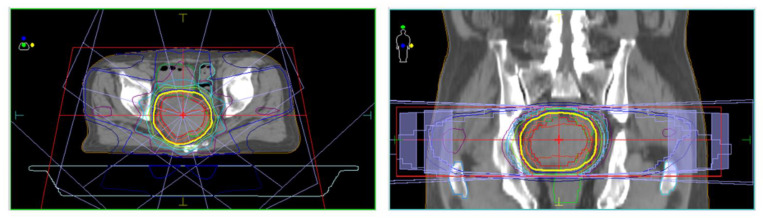
Treatment design of three-dimensional conformal radiation therapy (3D-CRT) for the metastatic tumor in the pouch of Douglas.

**Figure 6:**
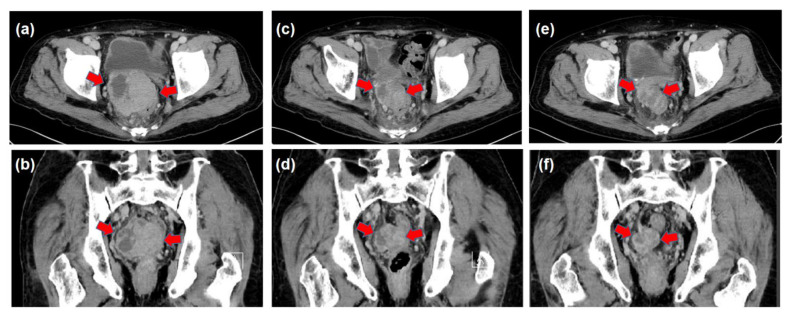
Before radiation therapy (RT), a large mass of heterogeneous density was observed ((**a**): Axial image and (**b**): Coronal image) (arrowheads). At three months after the completion of RT, the metastatic pelvic tumor was decreased in size ((**c**): Axial image and (**d**): Coronal image). The mass reduction was observed at the six months after the completion of the RT ((**e**): Axial image and (**f**): Coronal image).

**Figure 7:**
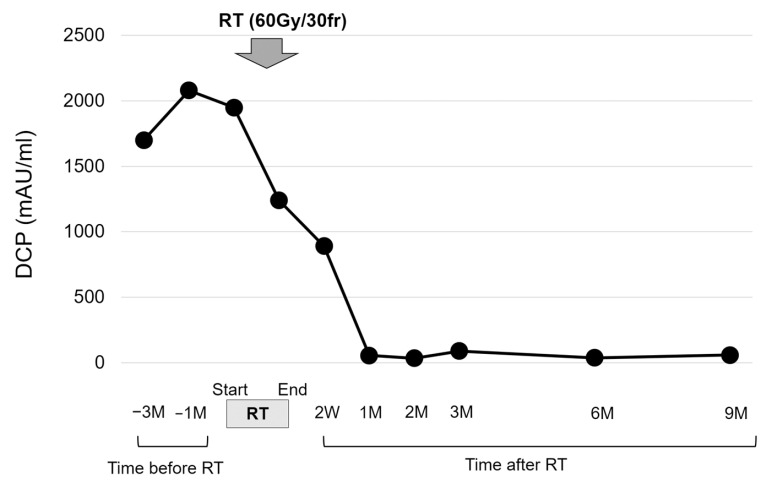
The transition in the des-gamma-carboxy prothrombin (DCP) values. The DCP value showed a marked decrease after radiation therapy (RT). The prescribed dose for the gross tumor was 60 Gy in 30 fractions (fr).

**Figure 8:**
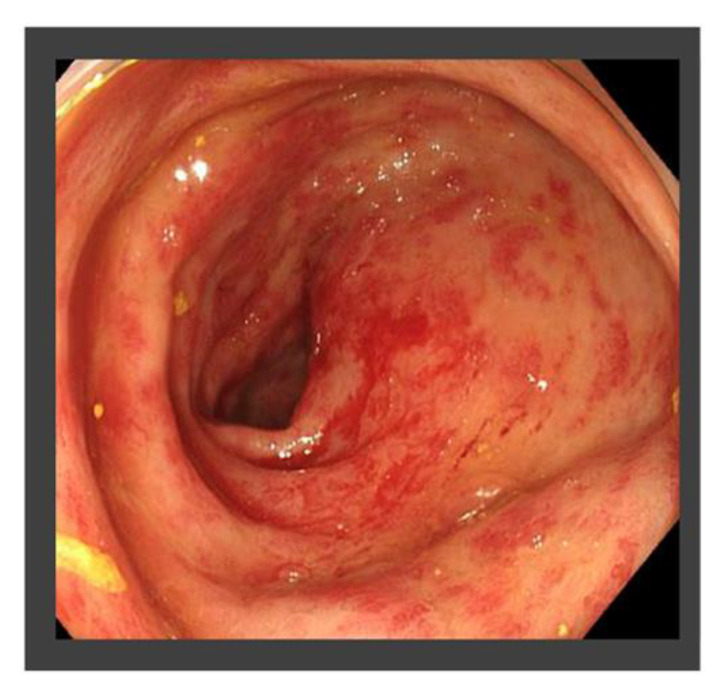
Colonoscopy findings at 9 months after radiation therapy (RT). Radiation-associated proctitis findings, including, telangiectasia, edema, erythema, and bleeding were observed.

The patient had suffered from diabetes and been diagnosed with liver cirrhosis due to nonalcoholic fatty liver disease. He had a history of ruptured hepatocellular carcinoma (HCC), located near the surface of the right posterior-inferior segment (Segment VI) of the liver, and had undergone emergent hemostasis with transcatheter arterial embolization (TAE). He had also received subsequent radiofrequency ablation for the curative treatment of HCC. When the HCC treatment had been completed, his liver reserve function had shown a decompensated status (Child–Pugh grade B). Because of the patient’s history of ruptured HCC and increased DCP value, we assumed the presence of metastasis in the pouch of Douglas. A medical interview revealed the presence of mild constipation, and the colonoscopy showed a normal rectal mucosa with compression by an extra-luminal tumor (Figure 3). Histological assessment with computed tomography-guided percutaneous needle biopsy revealed that the tumor was metastasis of HCC (Figure 4). He had decompensated cirrhosis (Table 2), and the application of surgical resection or systemic chemotherapy was considered difficult. The large pelvic tumor was the only recurrent lesion that we detected, and we conducted three-dimensional conformal radiation therapy (3D-CRT) with the aim of controlling the metastatic lesion (Figure 5). The prescribed dose for the gross tumor was 60 Gy in 30 fractions, and radiation therapy was successfully completed without any obvious adverse events. The tumor size and DCP value was found to be remarkably decreased (Figure 6 and Figure 7). His liver function did not change after RT for the pelvic tumor, and his constipation was resolved. No severe side effects were observed, although occasional blood stool due to radiation proctitis (grade 1) occurred from approximately 9 months after the completion of RT (Figure 8).

## 3. Discussion

In Japan, HCC is one of the major malignancies due to the high prevalence of viral hepatitis [6]. Tumor rupture and bleeding is one of the serious complications of HCC, and the incidence is reported to be relatively high in Asia and Africa, ranging from 3 to 26% in HCC patients [7,8]. The mortality rate of patients after the rupture of HCC is reported to be 25–75%; however, the prognosis is suggested to have improved in recent years [7,8,9].

Ruptured lesions are most commonly observed in the left lateral segment (Segments II and III) or right posterior-inferior segment (Segment VI) [10]. Various strategies, such as emergent hepatectomy or TAE are conducted as hemostatic treatment [7]. In the present case, the bleeding occurred in a tumor located in Segment VI, and because of the poor liver reserve function, hemostatic TAE was conducted in another hospital. Therefore, our case showed the typical clinical history of ruptured HCC.

The most unique point in this case was the performance of 3D-CRT for the treatment of metastasis in the pouch of Douglas. Peritoneal dissemination is reported to occur in 2–15% of HCC patients [1]. HCC rupture, diaphragmatic invasion, and lymph node metastasis are reported to be risk factors for peritoneal dissemination [2]. In addition, needle tract seeding due to puncture techniques, including biopsy or percutaneous ablation, has been implicated in peritoneal dissemination [1,2]. The prognostic impact of peritoneal dissemination is unclear, as the progression of the intrahepatic malignant lesions or liver failure would mainly be related to the prognosis in patients with advanced HCC. Recently, systemic treatments for HCC have advanced [11,12] and can be used for patients with extrahepatic lesions [4,5,11,12,13]. However, some reports have suggested that the resection of a disseminated tumor may have beneficial effects on the prognosis [14,15,16]. Some studies have also suggested the clinical utility of cytoreductive surgery in combination with other therapies [17,18]. Therefore, local treatment for a disseminated lesion may be beneficial in certain cases. In our patient, the intrahepatic malignant lesions were well controlled, while the disseminated lesion formed a large tumor. We therefore speculated that the disseminated lesion had the potential to affect the patient’s prognosis. On the other hand, due to the poor hepatic reserve function, neither surgical resection nor systemic chemotherapy appeared to be applicable in our case. The concept of ‘oligometastasis’, which is a clinical state of metastasis with limited metastatic capacity that can be effectively treated with local therapies, has been proposed [19]. The idea has been accepted in various types of solid malignancies and is proposed to be applicable to HCC as well [20]. However, uncertainty concerning the application of such a concept to HCC cases remains, and systemic treatment is still the standard of care for HCC. Our case report may provide some suggestive information and help enhance research regarding “oligometastasis” of HCC.

HCC is known to be sensitive to radiotherapy, and the efficacy of RT has been reported [21,22]. The colonoscopy showed a submucosal tumor without invasion into the lumen (Figure 3), and the risk of perforation was predicted to be low. The disseminated lesion was reduced in size, and the patient’s tumor marker levels were markedly reduced, despite the development of radiation-associated proctitis (grade 1) as a mild side effect. There are few reports on the successful application of RT for the treatment of retroperitoneal metastasis in HCC patients, and to our knowledge, successful RT to treat metastasis of HCC in the pouch of Douglas has not been reported. Our case suggests that RT may be a viable strategy for treating a disseminated lesion in the pouch of Douglas, particularly in cases with a poor hepatic reserve function.

We herein describe a case that was successfully treated with RT. However, several limitations associated with the present study, such as alternative treatments and future applications, warrant mention. The tumor microenvironment in HCC is strongly immunosuppressive, and immunotherapies based on the use of immune checkpoint inhibitors have provided great promise in the treatment of HCC [23,24]. Such new therapies may provide an effective treatment option for this case. In addition, since we obtained the tumor tissue by a percutaneous biopsy, we were able to identify another effective treatment using recently developed technologies, such as cancer genomic profiling [25,26]. Finally, our case report suggests that more precise and individualized approaches need to be tested in well-designed clinical trials. Unfortunately, however, it is not easy for us to propose how our report can lead to such advances in medicine.

## 4. Conclusions

We experienced a case of HCC metastasis in the pouch of Douglas for which RT was effective. RT might be a viable treatment for metastasis of HCC in the pouch of Douglas, particularly in cases with an insufficient hepatic reserve function.

## Figures and Tables

**Table 2:** The laboratory findings of the case.

WBC	2780	/μL	(4000–9000)	T-bil	1.6	mg/dL	(0.2–1.2)	PT-INR	1.28		(0.91–1.14)
RBC	320	×10^4^/μL	(410–550)	AST	61	U/L	(13–30)	PT%	62.9	%	(70–120)
Hb	11.2	g/dL	(13.0–17.0)	ALT	57	U/L	(10–42)				(46–260)
Hct	33.3	%	(35.0–51.0)	ALP	197	U/L	(38–113)	HBsAg	(-)		(-)
Plt	88	×10^3^/μL	(150–350)	γ-GTP	23	U/L	(13–64)	HBsAb	(-)		(-)
				Alb	2.3	g/dL	(4.1–5.1)	HCVAb	(-)		(-)
Na	137	mmol/L	(138–145)	NH3	146	μg/dL	(12–66)				
K	4.0	mmol/L	(3.6–4.8)					CEA	5.6	ng/mL	(≤5.0)
Cl	108	mmol/L	(101–108)	BS	180	mg/dL	(70–109)	CA19–9	16.3	U/mL	(≤37.0)
BUN	14	mg/dL	(8–20)	HbA1c	4.8	%	(4.6–6.2)	AFP	2.9	ng/mL	(≤10.0)
CRE	0.73	mg/dL	(0.65–1.07)	CRP	0.29	mg/dL	(≤0.3)	DCP	1950	mAU/mL	(<40)

CRE: creatinine, ALP: alkaline phosphatase, γ-GTP: γ-glutamyl transpeptidase, BS: blood sugar, CRP: C-reactive protein, CEA: carcinoembryonic antigen, CA19-9: carbohydrate antigen 19-9, AFP: α-fetoprotein, DCP: des-gamma-carboxy prothrombin.

## Data Availability

Data supporting this case report are available from the corresponding author on reasonable request.
